# Selective Catalytic Production of Urea: Unravelling the Pairwise Competition on *p‐d* Alloy Catalysts

**DOI:** 10.1002/advs.76725

**Published:** 2026-07-21

**Authors:** Qingchao Fang, Yun Han, Md Tarikal Nasir, Fuyong Qin, Wanhu Tian, Hanqing Yin, Aijun Du

**Affiliations:** ^1^ School of Petroleum and Chemical Engineering Dongying Vocational College Dongying China; ^2^ School of Chemistry and Physics Queensland University of Technology Brisbane Australia; ^3^ School of Chemistry and Physics QUT Centre for Materials Science Queensland University of Technology Brisbane Australia; ^4^ School of Physics and Electronic Information Weifang University Weifang China; ^5^ Department of Energy Technical University of Denmark Copenhagen Denmark

**Keywords:** DFT simulations, electrocatalysis, pairwise competition, *p‐d* alloys, urea synthesis

## Abstract

Constructing asymmetric structures facilitates electrocatalytic urea synthesis by enabling targeted reactant activation. However, accurate mechanistic understanding is complicated by intricate pairwise competitions among initial adsorption, C‐N coupling, and parasitic hydrogenation. Herein, by decoding these competitive routes on indium‐based coinage metals (In_4_M_9_, M = Au, Ag, and Cu), we systematically uncover an intrinsic activity sequence of Ag > Cu > Au. To rationally evaluate these disparate pathways, we deduce two quantifiable stage‐specific descriptors: the Anchoring Affinity (*S_anchor_*) for assessing robust reactant capture against proton attack, and the Coupling Propensity (*S_couple_*) for evaluating C‐N combination likelihood over fatal hydrogenation. A desired catalytic zone is defined from seven alloy systems under the quantitative criteria of *S_anchor_* < 0 and −0.3 < *S_couple_* < −0.12. Strikingly, In_4_Ag_9_ falls precisely into this desired zone, driving urea synthesis with a minimal limiting energy of only 0.31 eV. Electronic analyses reveal that In_4_Ag_9_ affords an appropriate NO activation, maintaining an ideal thermodynamic balance between NO reduction and C‐N coupling. Ultimately, this work unravels the atomic‐level pairwise mechanisms and provides straightforward, quantifiable descriptors as a powerful compass to guide targeted catalyst design.

## Graphical abstract

Unraveling the complicated competition in urea electrosynthesis, this work investigates asymmetric *p‐d* alloys. We establish two stage‐specific descriptors, Anchoring Affinity and Coupling Propensity, to elegantly quantify thermodynamic bottlenecks. Guided by these precise indices, In_4_Ag_9_ emerges within the optimal catalytic zone, driving urea production with a minimal 0.31 eV energy input owing to a “just‐right” NO activation.

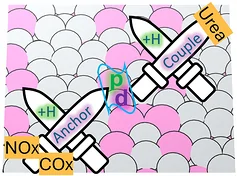

## 1 Introduction

Urea (CO(NH_2_)_2_) is an indispensable N‐containing raw material in the chemical industry and globally sustains agriculture as a high‐nitrogen fertilizer [1]. The classical Bosch‐Meiser urea process, however, demands energy‐intensive conditions (150–250 bar, >180°C) and triggers colossal CO_2_ emissions [2]. Driven by global decarbonization, electrocatalytic urea synthesis directly from ubiquitous small molecules at ambient conditions offers an immensely promising, sustainable alternative. Nevertheless, electrocatalytic C‐N coupling requires highly sophisticated proton‐coupled electron transfer (PCET) pathways [3]. The co‐existence of elusive C‐ and N‐containing intermediates inevitably incites parasitic side reactions, such as the reduction of CO_x_ into C1 hydrocarbons like CO and CH_4_, NO_x_ into N_2_ or NH_3_, and the hydrogen evolution reaction with similar reaction potential (HER, H^+^ to H_2_), which severely deplete the Faradaic Efficiency (FE) and target selectivity [4]. Therefore, deciphering the fundamental reaction preferences to effectively suppress these competitive pathways remains the paramount hurdle for efficient urea production.

For complex electrocatalytic processes involving multiple reactants (e.g., NO_x_ and CO_x_), constructing asymmetric catalytic sites has emerged as a compelling strategy to simultaneously accommodate and coordinate the diverse adsorption requirements of different intermediates [5, 6, 7, 8, 9, 10, 11, 12]. Recently, utilizing *p‐d* orbital coupling in bimetallic alloys, where transition metals are alloyed with main‐group elements, has become a highly sought‐after approach to create such asymmetric environments [13, 14, 15, 16, 17, 18, 19, 20, 21]. However, despite these geometric and electronic advantages, the electrocatalytic synthesis of urea on *p‐d* alloys remains a formidable challenge. Precise control over selectivity and Faradaic efficiency is constantly plagued by intricate competing networks, particularly the fierce competition among initial reactant adsorption, subsequent C‐N coupling, and parasitic intermediate hydrogenation [22, 23]. Traditionally, the adsorption energy of a single key intermediate constitutes a ubiquitous descriptor for reactant activation (e.g., ^*^NO, ^*^CO, and ^*^H) [24, 25, 26, 27]. Yet, on such multi‐site alloy surfaces under multi‐species co‐adsorption conditions, a single‐adsorbate energy descriptor falls remarkably short. It fails to accurately capture the dynamic, pairwise competition inherent in the multistep network, which severely limits its predictive power and hinders the rational design of high‐performance, selective electrocatalysts.

To bridge this mechanistic gap, this work proposes a stagewise quantification paradigm to evaluate the complex pairwise competition and directly guide the design of selective *p‐d* alloy catalysts. Specifically, we deconvoluted the urea synthesis network into two ubiquitous competitive stages: initial reactant adsorption versus parasitic hydrogenation (Stage 1), and intermediate coupling versus continued hydrogenation (Stage 2). Using experimentally available In‐based coinage metals (In_4_M_9_, M = Au, Ag, and Cu) as prototypical testbeds [28], we systematically mapped out their thermodynamic reaction pathways. Strikingly, we deduced two robust stage‐specific descriptors derived from basic binding energies to identify advantageous reaction profiles: the Anchoring Affinity (*S_anchor_*) for evaluating robust reactant capture against proton attack in Stage 1, and the Coupling Propensity (*S_couple_*) for determining the C‐N pairing likelihood over fatal hydrogenation in Stage 2. Driven by distinct *p‐d* hybridization modes, In_4_Ag_9_ ideally falls into the defined desired spatial zone (*S_anchor_* < 0 and −0.3 < *S_couple_* < −0.12), exhibiting seamless ^*^NO/^*^CO co‐adsorption and facile C‐N coupling with a low energy cost of 0.31 eV. Ultimately, this work not only elucidates the origin of *p‐d* alloys’ asymmetric reactivity but also provides the dual quantifiable criteria as a simplified yet powerful compass to predict and optimize targeted urea production.

## 2 Results and Discussion

The identification of specific reaction stages and critical intermediates is crucial for elucidating reaction mechanisms. The electrocatalytic urea synthesis involves a complicated network of adsorption, hydrogenation, and coupling steps encompassing various intermediates. As illustrated in the simplified reaction pathways in Figure 1, the fundamental prerequisite is the co‐adsorption of two NO and one CO molecules. However, a competition between reactant co‐adsorption and adsorbate hydrogenation poses a significant barrier to subsequent coupling and urea production. As is highlighted in the first green box (Stage 1), one adsorbed NO can be attacked by a proton‐electron pair rather than undergoing co‐adsorption with another NO or CO molecule. The situation continues for the third molecule of adsorption. If the co‐adsorption of these three molecules is successful, the reaction proceeds to the second stage, characterized by the competition between coupling and hydrogenation steps (highlighted in the orange box, Stage 2), which is complicated by the three adsorbates’ provision of six possible sites for hydrogenation and three kinds of coupling configurations (NO‐CO, NO‐NO, and CO‐another NO).

**FIGURE 1:**
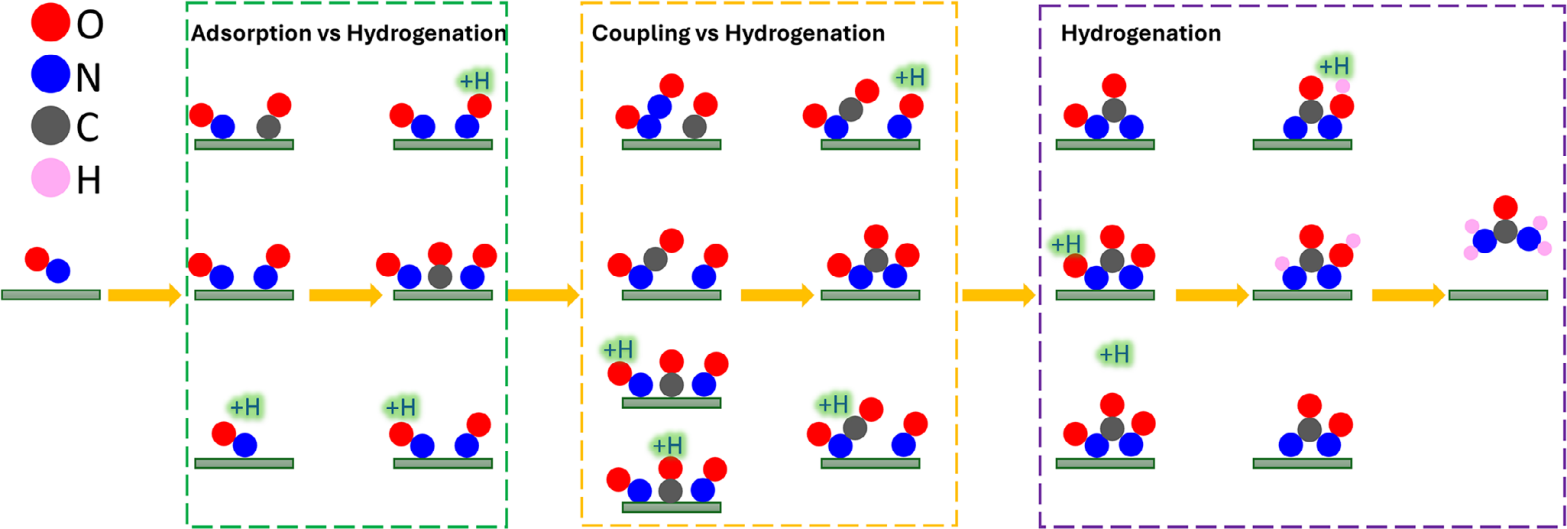
Schematic illustration of general reaction pathways for electrocatalytic urea production. The pathways are classified into three distinct stages featuring two primary competitive networks: NO/CO adsorption vs. hydrogenation (Stage 1), and the coupling of adsorbates C‐N/N‐N coupling vs. adsorbate hydrogenation (Stage 2).

In this context, the early‐stage role of hydrogenation is either neutral or detrimental, since excessive hydrogenation may lead to the formation of CH_4_, NH_3_, N_2_ or N_2_O by‐products. Likewise, two successful coupling steps of the adsorbates are essential. Once an intermediate featuring N─C─N bonds is formed, it may undergo subsequent hydrogenation steps as in the third stage (highlighted in the purple box). Successive protonation of the N atoms will ultimately lead to the formation of urea. If not, the preference for hydrogenation over adsorption/coupling may lead to the formation of C, N‐based byproducts. Therefore, elucidating this preference is crucial for the evaluation of catalytic performance and the rational design of advanced alloy catalysts. Additionally, to balance the computational cost associated with the extensive number of reaction steps and intermediate models, standard gas‐phase DFT calculations incorporating an implicit solvation model were utilized.

To translate these theoretical reaction stages into practical material evaluations, the selection of suitable candidates is vital both for evaluating catalytic performance and for elucidating the catalytic trends across multiple reduction steps. Herein, indium‐based coinage metal alloys (In_4_M_9_, where M = Au, Ag, and Cu) adopting the same γ‐brass type phase were selected (Table S1), all of which possess an identical valence electron concentration (≈1.61) according to the Hume‐Rothery electron concentration rule [29]. The defined stoichiometry and structural similarity ensure high stability and exclude the influence of disordered surface atomic arrangements, aiding the establishment of a well‐controlled model system. The constructed models are expected to exhibit similar surface geometries with varied electronic structures, which effectively eliminates geometric effects on the catalysts’ performance. Furthermore, these alloys have already been experimentally synthesized, underscoring their potential for practical catalytic applications. As shown in Figure 2a–c, the most stable low‐index facet was optimized to investigate NO adsorption. Given the high stoichiometric ratio of the coinage metals, a majority of the exposed surface atoms are M atoms. Various possible adsorption configurations were evaluated, and the most stable configuration involves NO binding to the surface via the N atom (Figure S1). NO adsorption at single‐atom sites (on top of In and coinage metals) is unfavorable with a higher Gibbs free energy.

**FIGURE 2:**
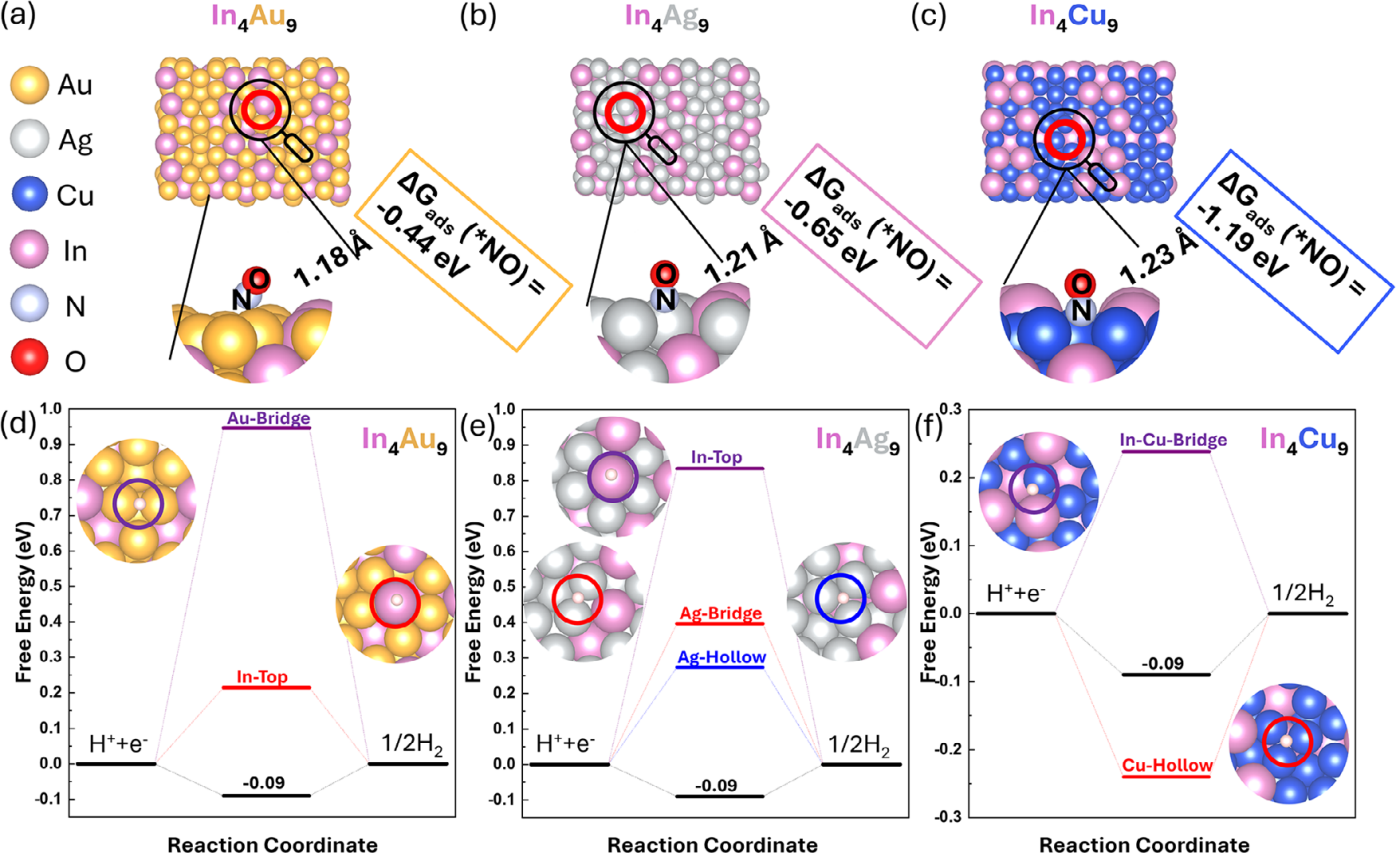
Optimized slab configurations of the three In‐based electrocatalysts. (a) In_4_Au_9_, (b)In_4_Ag_9,_ and (c) In_4_Cu_9_, with magnified views of the thermodynamically favorable active sites. The corresponding nitric oxide (NO) adsorption configurations, Gibbs free energy (ΔG_ads_(^*^NO)), and N─O bond lengths are shown below each structure. (d–f) Free energy diagrams for proton adsorption on various sites of In_4_Au_9_, In_4_Ag_9_, and In_4_Cu_9_. The insets denote the top views of the sites considered for proton adsorption. The black line displays the commercial Pt|C catalyst for reference.

The Gibbs free energies of NO adsorption onto the Au‐, Ag‐, and Cu‐based slabs are −0.44 eV, −0.65 eV, and −1.19 eV, and the corresponding N─O bond lengths are 1.18 Å, 1.21 Å, and 1.23 Å, respectively. These results indicate the effective capture and activation of the adsorbate. Simultaneously, the competing HER was also evaluated to assess catalyst selectivity. The hydrogen adsorption free energy (ΔG_*H_) is a widely accepted descriptor for the evaluation of HER performance. However, in the context of urea electrosynthesis, a ΔG_*H_ value close to 0 eV implies a highly active HER side reaction, which inevitably leads to low Faradaic Efficiency (FE) and poor selectivity for urea production. Hydrogen adsorption on various sites was examined (Figure 2d–f), revealing that ^*^H preferentially binds to the In‐top, coinage metal‐bridge, and hollow sites. Compared to the commercial Pt/C catalyst (−0.09 eV), the hydrogen adsorption free energies of these three catalysts deviate significantly from the ideal HER value (0 eV). This substantially suppresses HER activity, thereby ensuring excellent selectivity towards urea production.

To elucidate the reaction mechanism on In_4_Au_9_, the thermodynamically favorable reaction pathway, along with various intermediates, was identified based on the Gibbs free energy changes (ΔG). The preference for a specific intermediate at each bifurcation point is determined by the free energy difference. As shown in Figure 3a and Figure S2, the Gibbs free energy profiles of NO reduction and the corresponding intermediates are summarized across different stages (labeled 1 to 6). The magenta symbols denote the thermodynamically favorable intermediates, while the grey ones are excluded from subsequent steps due to their relatively higher free energies. After the initial NO adsorption (No.2), the formation of ^*^NHO, ^*^NO^+*^CO, and ^*^NO^+*^NO is favored with free energy changes of −0.01 eV, −0.17 eV, and −0.89 eV, respectively, while ^*^NOH formation is relatively unfavorable (ΔG = 0.32 eV, No.3). This indicates that, initially, NO adsorption is favored over hydrogenation. However, the ^*^NO^+*^NO intermediate is preferentially reduced to ^*^NO^+*^N, bypassing CO co‐adsorption or protonation on the N atom (No.4–No.5). Subsequent protonation of the oxygen atom in the adjacent NO molecule (No.6) triggers spontaneous N‐N coupling, resulting in the release of gaseous N_2_. Evidently, in Stage 2, N─N coupling outcompetes the hydrogenation of N‐containing intermediates, which is supported by the preferential formation of N_2_ instead of NH_3_.

**FIGURE 3:**
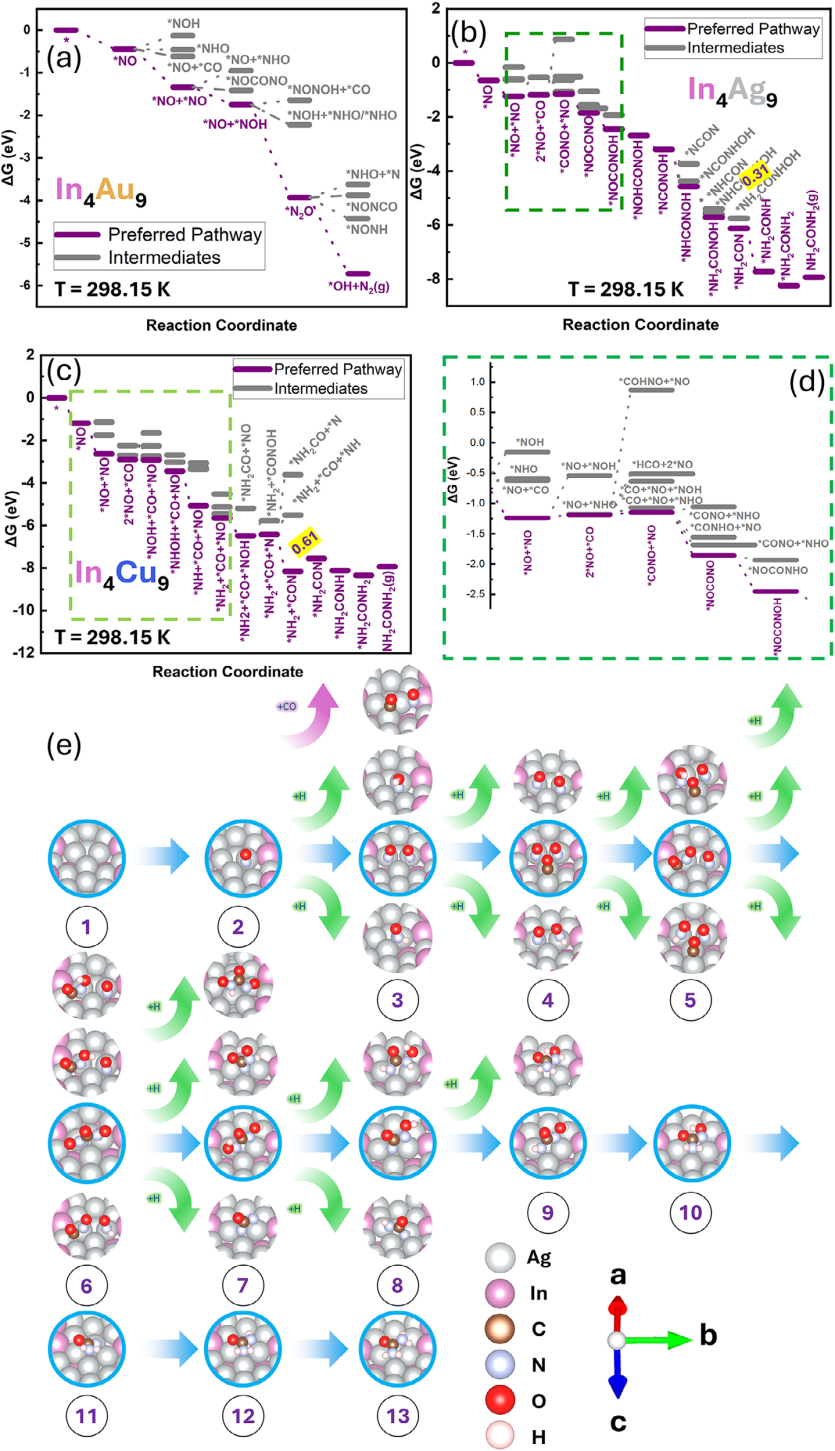
Gibbs free energy diagrams for urea electrosynthesis on (a) In_4_Au_9_, (b) In_4_Ag_9_, and (c) In_4_Cu_9_ along the optimal reaction pathways. Thermodynamically favorable intermediates are denoted in magenta, while the unfavorable ones are marked in grey. (d) A magnified view of the specific reaction stages from ^*^NO^+*^NO to ^*^NOCONO in the upper green box. (e) The optimized configurations of key intermediates on In_4_Ag_9_. The thermodynamically favorable pathway is indicated by blue arrows. Competition between CO adsorption and adsorbate hydrogenation is also highlighted by the magenta and light green curved arrows, respectively.

In contrast to the N─N coupling observed on Au, a different catalytic pathway for urea production was found on the In_4_Ag_9_ (Figure 3b, with an enlarged view of specific reaction stages provided in Figure 3d for clarity). The corresponding intermediates are shown in Figure 3e. In Stage 1 (steps No.3 to No.4), the sequential adsorption of NO and CO is preferred, with Gibbs free energy changes of −0.59 eV and 0.05 eV, respectively. Protonation at either the O or N atom is thermodynamically unfavorable. In Stage 2, the C─N coupling of ^*^CO and ^*^NO (ΔG = 0.04 eV) is favored over the hydrogenation steps (No.5), and the second C─N coupling with another ^*^NO is also preferred (ΔG = −0.71 eV, No.6). The formation of the critical ^*^NOCONO intermediate establishes the structural framework of the urea molecule. After seven successive hydrogenation steps, the two remaining oxygen atoms attached to the N atoms are easily removed (ΔG < 0) as water molecules, followed by the desorption of the urea molecule, requiring a free energy change of 0.31 eV. Thus, for both stages in the In_4_Ag_9_ system, the co‐adsorption of NO and CO is preferred over the hydrogenation steps. Afterwards, the facile coupling of ^*^CO with two ^*^NO molecules still outcompetes hydrogenation, which ultimately drives the formation of urea after deoxygenation steps. This reaction preference for adsorption and C─N coupling over hydrogenation makes In_4_Ag_9_ a highly promising candidate for urea production.

Similarly, for the In_4_Cu_9_ system, the thermodynamically favorable intermediates along the optimal reaction pathway were also demonstrated, and the corresponding configurations are shown in Figure 3c and Figures S3 and S4. In Stage 1 (steps No.3 and No.4), the competition between adsorption and hydrogenation exhibits a similar trend to that of the In_4_Ag_9_ system. Herein, the adsorption of the second NO and CO (with ΔG of −1.43 eV and −0.27 eV, respectively) is favored over the hydrogenation steps, providing the essential precursors for the subsequent coupling process. However, in Stage 2 (steps No.5 to No.10), the hydrogenation steps (indicated by light blue and green arrows) are always preferred over the C‐N coupling steps (indicated by light yellow arrows). The second C─N coupling of ^*^NH_2_ and ^*^CON requires a ΔG of 0.61 eV, serving as the potential‐determining step (PDS) for the overall process. For the final desorption, an energy penalty of 0.41 eV is demanded for the release of the urea molecule, making it thermodynamically challenging to synthesize the desired product. Thus, it can be concluded that despite the adsorption preference over hydrogenation in Stage 1, the preference for hydrogenation over coupling in Stage 2 severely impedes the formation of the N─C─N bond and increases the risk of byproduct formation. The relatively high energy demands of the PDS and the large desorption energy make In_4_Cu_9_ less competitive than the In_4_Ag_9_ candidate. Besides, the C─N coupling steps for In_4_Ag_9_ and In_4_Cu_9_ were also examined, as shown in Figure S8. It can be concluded that the catalytic performance of In_4_Ag_9_ is superior to that of In_4_Cu_9_ due to its lower energy barriers. For the effects of solvent and pH in Figure S9, In_4_Ag_9_ still exhibits good performance with low limiting potential (0.24 eV) and energy changes (0.15–0.57 eV) for urea synthesis as compared to published work [30, 31].

To elucidate the underlying origins of the observed catalytic activity trend, electron localization function (ELF), partial density of states (PDOS), and Bader charge analyses were conducted (Figure 4). For NO adsorption on the three catalyst surfaces, the corresponding ELF maps are displayed in Figure 4a. Red areas show high electron localization with large ELF values, while the blue areas indicate minimal electron localization or depleted electron density. Significant electron redistribution is evident, with pronounced electron localization around the adsorbed NO and reduced electron density around the reactive metal sites (Au, Ag, and Cu atoms). PDOS analysis in Figure 4b shows that the d orbitals of Ag exhibit strong orbital overlap with the *p* orbitals of NO. Furthermore, the charge accumulation and depletion regions upon NO adsorption onto the surfaces are illustrated in Figure 4c. It can be seen that all surfaces act as electron reservoirs and contribute varying amounts of electrons to the adsorbed NO (0.08, 0.40, and 0.58 e^−^). The amount of electron transfer is in good agreement with the adsorption free energies of NO, as illustrated in Figure S5. Additionally, the work functions and PDOS of the three catalysts are compared (Figures S6 and S7). The work function trend indicates a strong electron‐binding affinity of the In_4_Au_9_ surface, while In_4_Ag_9_ and In_4_Cu_9_ surfaces demonstrate strong electron‐donating abilities. Significant differences also exist in the orbital interactions between the transition metal and In atoms. The *d* orbitals of Au exhibit pronounced hybridization with the *p* orbitals of In near the Fermi level, corresponding to stronger Au‐In bonding, while the hybridization between the Ag/Cu *d* and In *p* orbitals is comparatively weaker. Thus, the electronic states near the Fermi level are modulated by the *p‐d* hybridization in the In_4_Au_9_ and In_4_Ag_9_ systems. This moderate *p‐d* orbital hybridization may facilitate intermolecular adsorption, C−N coupling, and hydrogenation in the subsequent reaction steps.

**FIGURE 4:**
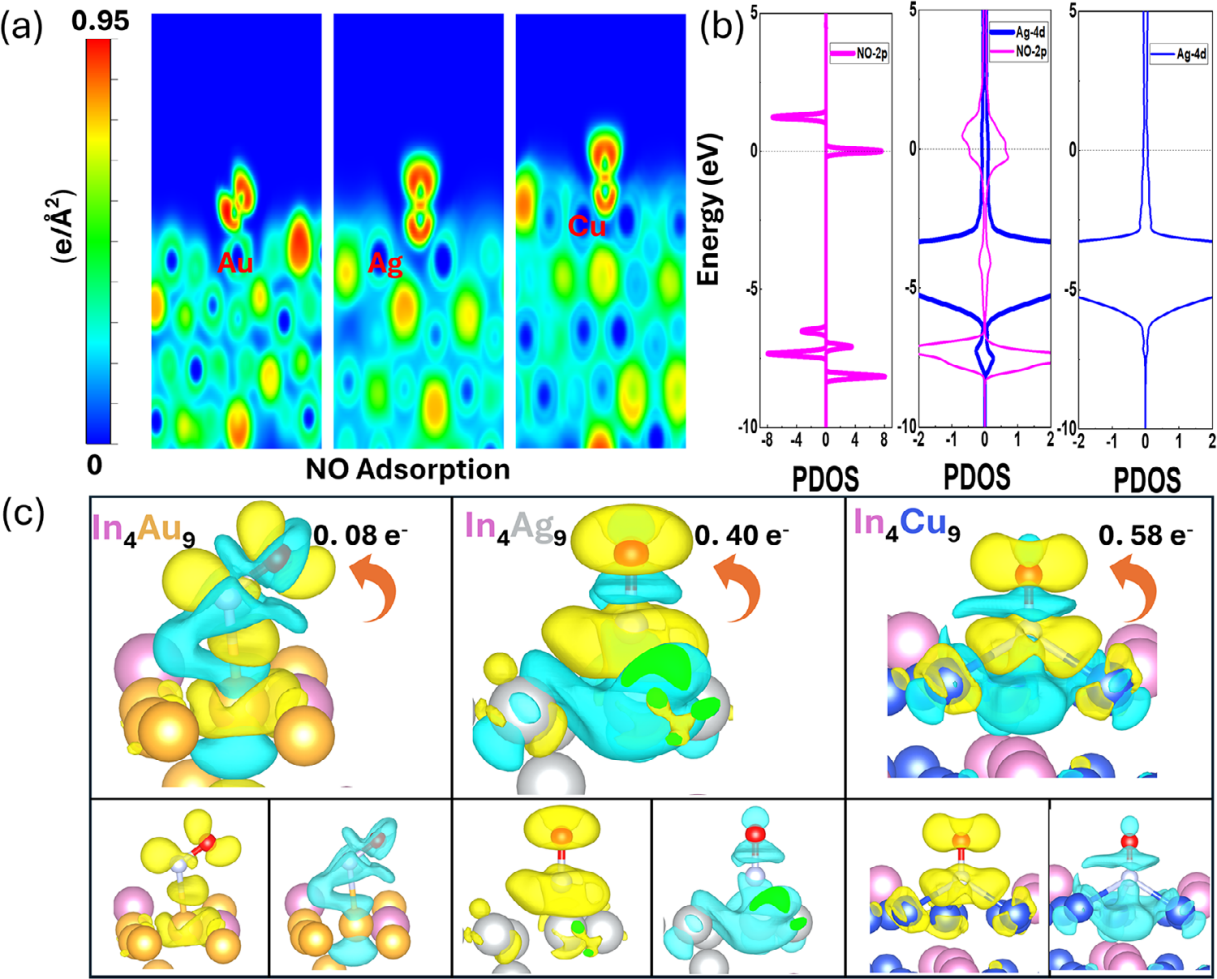
(a) Electron localization function (ELF) contours for NO adsorbed onto the In_4_Au_9_, In_4_Ag_9_, and In_4_Cu_9_ surfaces, respectively. (b) Partial density of states (PDOS) comparisons among the *p* orbitals of isolated NO, NO adsorbed on the In_4_Ag_9_, and the pure In_4_Ag_9_ slab. (c) charge density difference maps of the adsorbed NO and In_4_Au_9_, In_4_Ag_9,_ and In_4_Cu_9_ surfaces. The yellow and cyan areas represent electron accumulation and depletion, respectively. The iso‐surface level is set to 0.001 e bohr^−3^.

Evaluating catalyst candidates for urea production is highly challenging due to the multiple reaction steps involving various intermediates. As can be concluded from above, the reaction mechanism can be roughly classified into two competing stages: (i) adsorption vs. hydrogenation (Stage 1) and (ii) coupling vs. hydrogenation (Stage 2). For the In_4_Au_9_ system, spontaneous N─N coupling can convert NO into N_2_ instead of NH_3_, highlighting the potential risk of excessively strong coupling. Conversely, In_4_Cu_9_ demonstrates the consequences of hydrogenation over coupling. This preference can continuously reduce N_x_O_y_ intermediates into N_x_H_y_ species with postponed C─N coupling. In contrast, In_4_Ag_9_ emerges as the superior catalyst for urea production due to its well‐balanced thermodynamics across the adsorption, coupling, and hydrogenation steps.

Given that urea production from NO and CO is a highly complex process with multiple intermediates at every step (resulting from adsorption, coupling, and hydrogenation), it provides an extensive network of energetic variations to be investigated. As shown in Tables S2–S4, the practical competitiveness of In_4_M_9_ (M = Au, Ag, and Cu) systems was demonstrated by adsorption vs hydrogenation and two coupling vs hydrogenation stages. The thermodynamically favourable intermediates in adsorption and coupling promote critical bonding of N─CO─N for urea synthesis. To fundamentally quantify this pairwise competition, we employed the well‐established principle that the reaction energies of intermediates scale linearly with the adsorption energies of key species. Accordingly, two descriptor metrics, the initial Anchoring Affinity (*S_anchor_*) for Stage 1 and the Selective Coupling Propensity (*S_couple_*) for Stage 2, were derived through ordinary least squares (OLS) fitting based on the fundamental adsorption free energies of NO, CO, and H:

(1) $$S_{anchor}=0.79E_{NO}+0.17E_{CO}-0.03E_{H}$$

(2) $$S_{couple}=-0.44E_{NO}+1.35E_{CO}-1.34E_{H}$$

The applicability of two descriptor metrics was also expanded to other alloy systems (Al_4_Cu_9_, Ga_4_Cu_9_, Ag_3_Sn, and InPt_3_) whose free energy evolution was shown in Figure S10.

As illustrated in Figure 5a, a 2D activity map representing the reaction preferences is constructed based on *S_anchor_* and *S_couple_*, displaying the relative positions of the studied coinage metals. The blue line (*S_anchor_* = 0) indicates the thermodynamic balance between NO/CO adsorption and competitive hydrogenation (Stage 1). The region to the left of this boundary indicates a thermodynamic preference for NO/CO adsorption, which is a prerequisite for subsequent steps. The area above the red line (*S_couple_* = −0.12) indicates that hydrogenation is favored over C─N coupling (Stage 2), while the opposite trend dominates in the area below the green line (*S_couple_* = −0.30). Thus, the light‐yellow area represents the optimal target region for both stages. Within this zone, NO/CO can be spontaneously adsorbed (in Stage 1) and possesses a higher probability of being coupled rather than forming NH_3_ or N_2_ byproducts (in Stage 2). For all the alloy systems, the *S_anchor_* values are consistently below 0, indicating favorable adsorption of NO/CO. The *S_couple_* can be grouped by light pink, light yellow, and light green, which denotes excessively strong coupling (light green zone) and dominant hydrogenation (light pink zone). Conversely, the moderate *S_couple_*, ranging from −0.30 eV to −0.12 eV, indicates an optimal balance between coupling and hydrogenation, thereby aiding the formation of key N─C─O intermediates for urea synthesis. As shown in Figure S11, the highest C─N coupling energy change of alloys was summarized. From the upper zone to the lower one, the energy required for C─N coupling becomes less and less, which reflects the trend from hydrogenation being favourable to coupling being favorable for different alloy systems.

**FIGURE 5:**
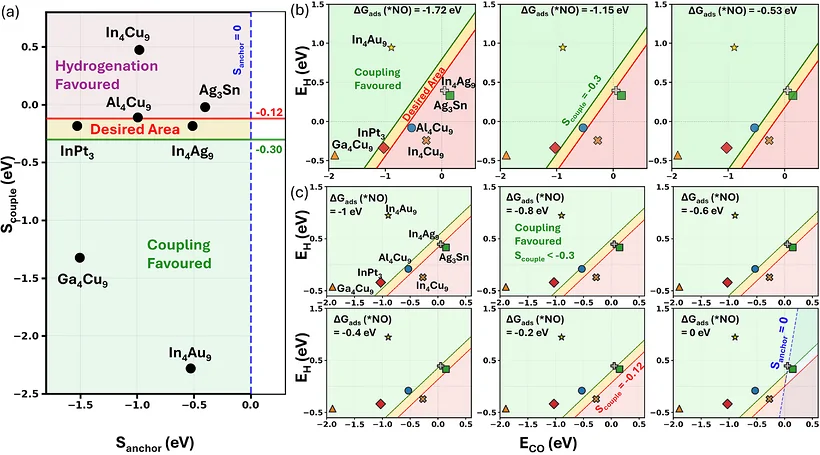
(a) A 2D selectivity map defined by *S_anchor_* and *S_couple_*. The region to the left of the blue line (*S_anchor_* = 0) denotes a preference for NO/CO adsorption over adsorbate hydrogenation. The regions above the red line (*S_couple_* = −0.12) and below the green line (*S_couple_* = −0.3) indicate preferences for adsorbate hydrogenation and adsorbate C─N coupling, respectively. The optimal target region (light‐yellow area) indicates favorable NO/CO adsorption coupled with a balanced compromise between C─N coupling and hydrogenation. The relative positions of seven candidates were plotted accordingly. (b) Positional shifts of seven candidates relative to the optimal target band under fixed NO adsorption energy, highlighting a theoretical catalytic performance trend regulated by NO adsorption energy. (c) Dynamic evolution of the optimal target area and the relative positions of seven candidates as the NO adsorption energy is systematically tuned from −1 eV to 0 eV.

To examine the interplay between CO and proton adsorption energies, the positional shifts of the three candidates under a fixed NO adsorption energy are plotted in Figure 5b. It can be seen that In_4_Au_9_ and Ga_4_Cu_9_ are far from the optimally desired region, and In_4_Cu_9_ is in the light pink zone (hydrogenation favourable zone). To further elucidate this trend, the NO adsorption energy was systematically varied from −1 eV to 0 eV, as shown in Figure 5c. The results verify that In_4_Au_9_ remains an unpromising catalyst because of its excessively strong coupling preference, regardless of the variation in NO adsorption energy. Meanwhile, as the optimal band moves obliquely downward, it causes many alloys, such as In_4_Ag_9,_ Ag_3_Sn, Al_4_Cu_9_, and In_4_Cu_9_, to gradually enter or exit the target zone. Notably, when the NO adsorption energy is tuned from −0.8 to −0.4 eV, the theoretical catalytic performance of In_4_Cu_9_ is significantly improved.

Additionally, the interplay of proton and NO adsorption energies as a function of varying CO adsorption energy is presented in Figure S12. Here, the target band shifts toward the upper right, rendering In_4_Ag_9_ and Ga_4_Cu_9_ promising at a CO adsorption energy of 0 eV, and In_4_Cu_9_ competitive in the range of −0.8 eV to −0.6 eV. However, the optimal band shifts toward the upper left with varying proton adsorption energies, as shown in Figure S13. In this scenario, Al_4_Cu_9_ falls within the optimal region when the proton adsorption energy is in the range of −0.2 eV to 0 eV. Thus, it can be concluded that the two competing stages are greatly influenced by the adsorption energies of NO, CO, and H. Through the rational adjustment of these binding strengths, the catalytic performance of the alloy candidates can be systematically optimized for urea production.

## 3 Conclusion

In summary, employing first‐principles calculations, we systematically investigated the electrocatalytic synthesis of urea over asymmetric *p‐d* alloy surfaces (In_4_M_9_, where M = Au, Ag, Cu). The intrinsic catalytic performance follows the sequence of Ag > Cu > Au, fundamentally governed by their distinct abilities to navigate complex, pairwise reaction competitions. Specifically, for the Au‐based alloy, the urea pathway is fatally intercepted by highly favorable N─N coupling, predominantly yielding gaseous N_2_. For the Cu‐based alloy, aggressive protonation easily outperforms C─N coupling, leading to excessive hydrogenation by‐products. Conversely, In_4_Ag_9_ facilitates a seamless thermodynamic pathway for both stable reactant co‐adsorption and subsequent C─N coupling, ultimately driving the entire urea production with an exceptionally low limiting energy input of 0.31 eV.

Electronic structure analyses, including PDOS, ELF, and charge density distributions, elucidate that the superior activity of In_4_Ag_9_ originates from the distinct charge transfer at the asymmetric In‐Ag sites. This specific *p‐d* hybridization endows the surface with an appropriate activation behavior toward NO, neither too weak to lose capture nor too strong to trigger over‐reduction, thereby locking in an ideal thermodynamic balance for ongoing C─N coupling.

To generalize these mechanistic insights and bridge the gap between phenomenological observation and rational design, we parameterized the competing relationships into two quantifiable, stage‐specific descriptors deduced from fundamental adsorption energies: the Anchoring Affinity (*S_anchor_*) for assessing stable reactant capture against initial proton attack (Stage 1), and the Coupling Propensity (*S_couple_*) for evaluating C‐N pairing likelihood against continued hydrogenation (Stage 2). By plotting these indices, we clearly demarcated an optimal catalytic zone (*S_anchor_* < 0, −0.3 < *S_couple_* < −0.12) wherein In_4_Ag_9_ perfectly resides.

Ultimately, this work goes beyond validating In_4_Ag_9_ as a highly promising electrocatalyst. By decoupling the complicated multi‐step synthesis into identifiable reaction blocks and translating them into two simplified thermodynamic indices, we provide a robust, quantitative compass. This mechanism‐driven paradigm sets clear boundaries to rationally direct surface adsorption adjustments, thereby accelerating the discovery and performance enhancement of targeted urea electrocatalysts.

## 4 Computational Details

The spin‐polarized first‐principles calculations were performed based on density functional theory (DFT) using the Vienna ab initio Simulation Package (VASP) [32, 33]. The ion‐electron interaction was treated by projector‐augmented wave (PAW) pseudopotentials [34]. The exchange‐correlation was described by the Perdew‐Burke‐Ernzerhof (PBE) functional [35, 36]. To incorporate the effects of van der Waals interactions, Grimme's DFT‐D3 method was adopted [37, 38]. A cutoff energy of 450 eV was used during the simulations. The Monkhorst‐Pack meshes were employed to sample the first Brillouin zone for the total energy calculation. The convergence criteria of energy and force were set as 0.01 eV/Å and 10^−5^ eV, respectively. The supercell size was expanded to ensure the unnecessary interactions between periodic images, and a vacuum layer of more than 16 Å was introduced along the z direction. The electron distribution analysis was conducted by the Bader charge population method as implemented by the Henkelmann group [39]. The computational hydrogen electrode (CHE) model was utilized for the calculations of free energy change, and the corrections, including zero‐point energy and entropy contribution, were included [40]. The VESTA package was used to display the optimized models and charge density [41]. An implicit solvation model implemented in VASPsol with a dielectric constant of 80 was employed to observe the influence of the solvent [42].

## Author Contributions

**Qingchao Fang**: software, formal analysis, Writing – original draft, data curation, investigation, validation, visualization. **Fuyong Qin**: investigation, validation, writing – review and editing. **Wanhu Tian**: validation, investigation, writing – review and editing. **Yun Han**: methodology, software, validation, investigation, writing – review and editing, supervision, visualization. **Aijun Du**: supervision, resources, project administration, writing – review and editing, conceptualization, methodology, funding acquisition. **Md Tarikal Nasir**: writing – review and editing, validation, investigation. **Hanqing Yin**: investigation, validation, writing – review and editing.

## Funding

A.D. greatly acknowledges funding support by the Australian Research Council (DP250104479, DP260104198).

## Conflicts of Interest

The authors declare no conflicts of interest.

## Supporting information




**Supporting File**: advs76725‐sup‐0001‐SuppMat.docx.

## Data Availability

The data that support the findings of this study are available in the supplementary material of this article.
