# Supplementary material for: Selective Catalytic Production of Urea: Unravelling the Pairwise Competition on p‐d Alloy Catalysts
Source: Adv Sci (Weinh). 2026 Jul 21:e76725. Online ahead of print. doi: 10.1002/advs.76725 (PMC13387030; doi:10.1002/advs.76725)
Supplement: Supplementary file 1 — Supporting File: advs76725‐sup‐0001‐SuppMat.docx. [file ADVS-9999-e76725-s001.docx]

Supporting Information

Selective Catalytic Production of Urea: Unravelling the Pairwise Competition on *p-d* Alloy Catalysts

Qingchao Fang, Yun Han^*^, Md Tarikal Nasir, Fuyong Qin, Wanhu Tian, Hanqing Yin, Aijun Du^*^

**Computation details**

The Gibbs free energy change ΔG for the reaction step during the urea production is calculated as follows[1]:

|  | $\Delta G= \Delta E+ \Delta E_{ZPE}-T\Delta S$ | (S1) |
| --- | --- | --- |

where $\Delta E$ is the energy difference between the energies of the intermediates calculated from the DFT calculations at 0K, ΔE_ZPE_ means the zero-point energy. T is 298.15 K and ΔS is the change of entropy. VASPKIT package is employed to compute the zero-point energy and entropies of the intermediates. Besides, the entropy and enthalpy of the gaseous molecules were also calculated from JANF thermodynamic tables using the VASPKIT package.[2] The chemical potential of (H^+^ + e^-^) at standard conditions equals 1/2* $\text{G}_{\text{H}_{\text{2}}}$ – neU, where U is the electrode potential relative to the RHE, n stands for the number of electrons involved in the reaction pathway. T = 298.15K and pH=0 at standard conditions.

The NO adsorption energy was also calculated based on the expression below:

|  | $E_{ads}= E_{slab-NO}- E_{slab}-E_{NO}$ | (S2) |
| --- | --- | --- |

where E_slab-NO_ represents the energies of NO adsorbed on the slab, E_slab_ stands for the computed energy of catalyst slabs and E_NO_ is the energy of gaseous NO.

To capture the trends of relative competition between adsorption, hydrogenation, and coupling, all fitting data are derived from DFT calculations performed in this work and the calculated adsorption energies of NO, CO and H were used as the input variables for constructing the descriptors. The sample size (n = 7) is utilized with a range of different chemical composition for descriptor evaluation. The R² values (0.61 and 0.56 for S_anchor_ and S_couple_, respectively) indicate that the descriptors capture the dominant trends. The MAE values (0.264 eV and 0.492 eV) are comparable to the intrinsic error of DFT calculations, confirming that the descriptors provide quantitatively meaningful predictions for trend prediction. To assess the generalizability of the descriptors and rule out overfitting, we performed leave-one-out cross-validation (LOOCV). The LOOCV results (0.54 and 0.44) confirm that the descriptor system is not overfitted to the seven training surfaces.

**
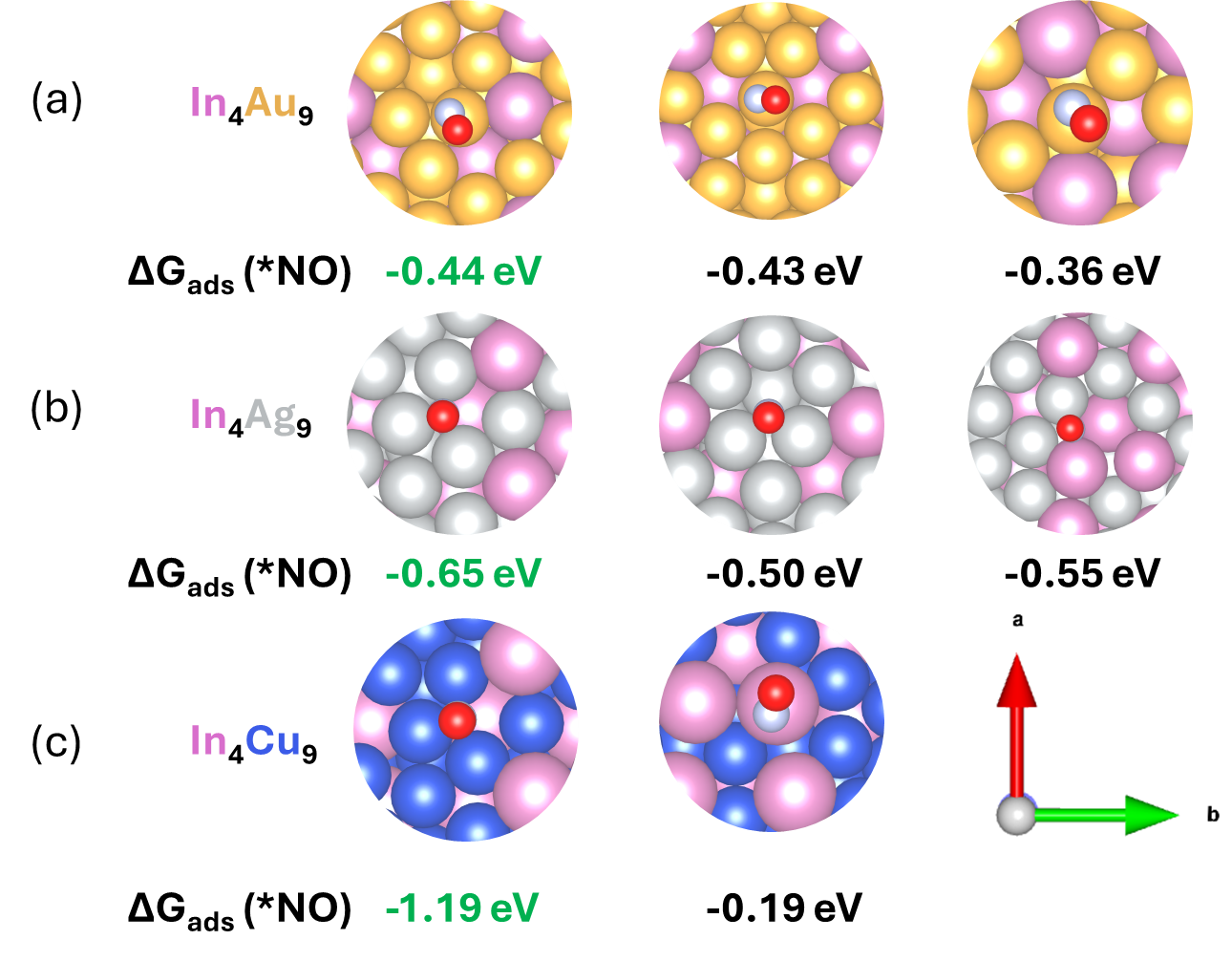
**

**Figure S1**. The NO adsorption free energy of In_4_Au_9_, In_4_Ag_9_, and In_4_Cu_9_ are displayed, where different NO configurations are attached with free energy below. The thermodynamically favourable ones are in green with the lowest free energy.


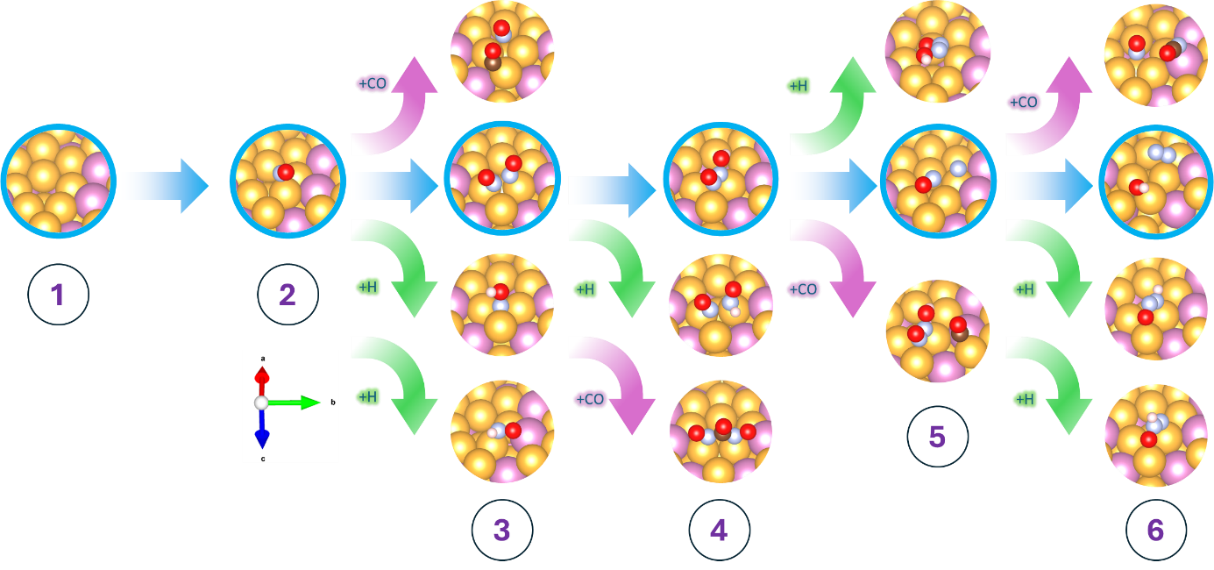


**Figure S2**. The optimized intermediate configurations of In_4_Au_9_ were displayed with the thermodynamically favorable pathway indicated by blue arrows. The CO adsorption vs adsorbate hydrogenation was also shown by the magenta and light green curved arrows.

**Figure S3**. The detailed magnified view of the partial reaction stage from *NO+*NO to *NH_2_+*CO+*NO for In_4_Cu_9_.


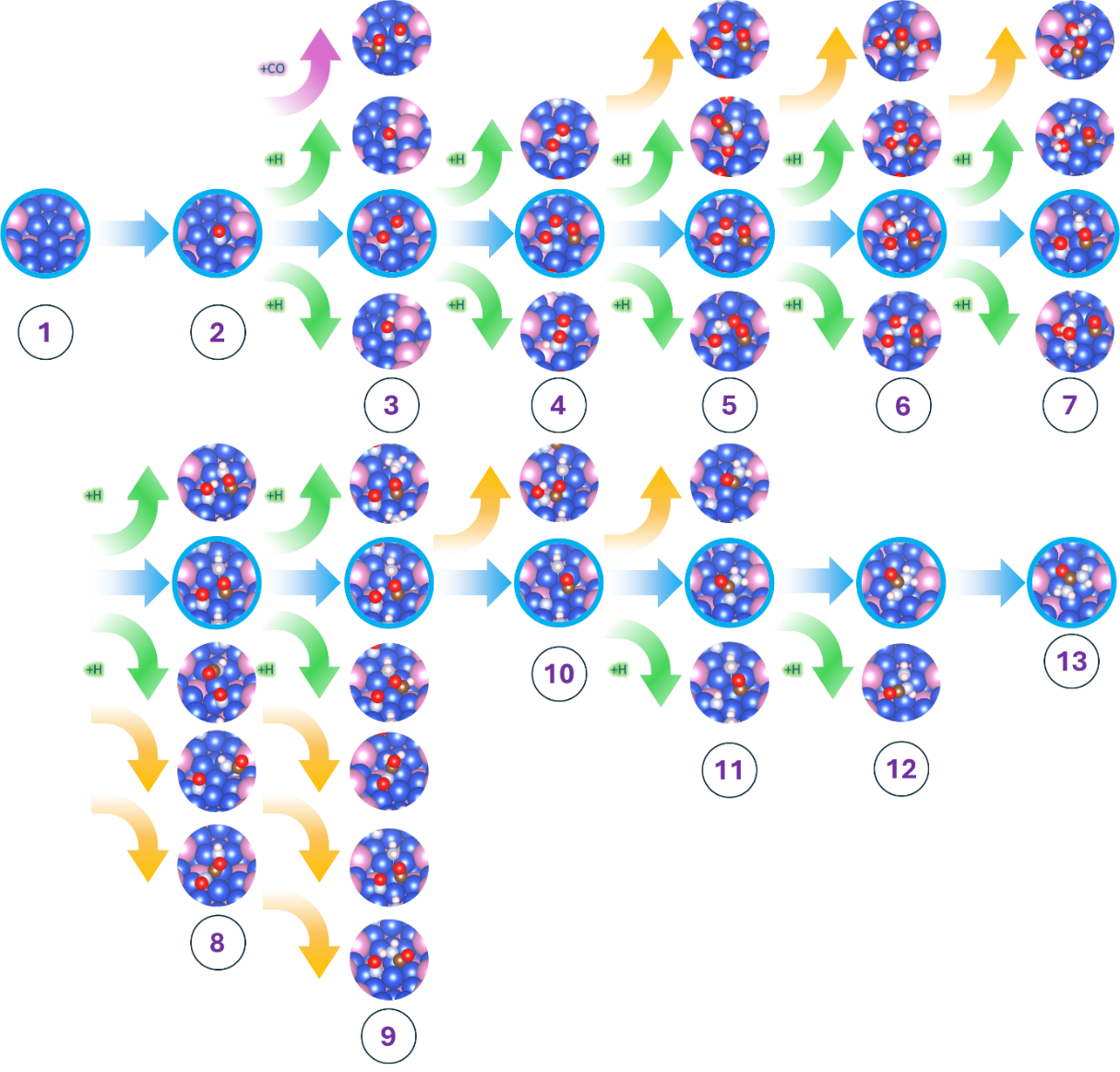


**Figure S4**. The optimized intermediate configurations on In_4_Cu_9_ were displayed with the thermodynamically favourable pathway indicated by blue arrows. The CO adsorption vs adsorbate hydrogenation was shown by the magenta and light green curved arrows. Similarly, the adsorbate coupling vs hydrogenation step was shown by the light yellow and green curved arrows.


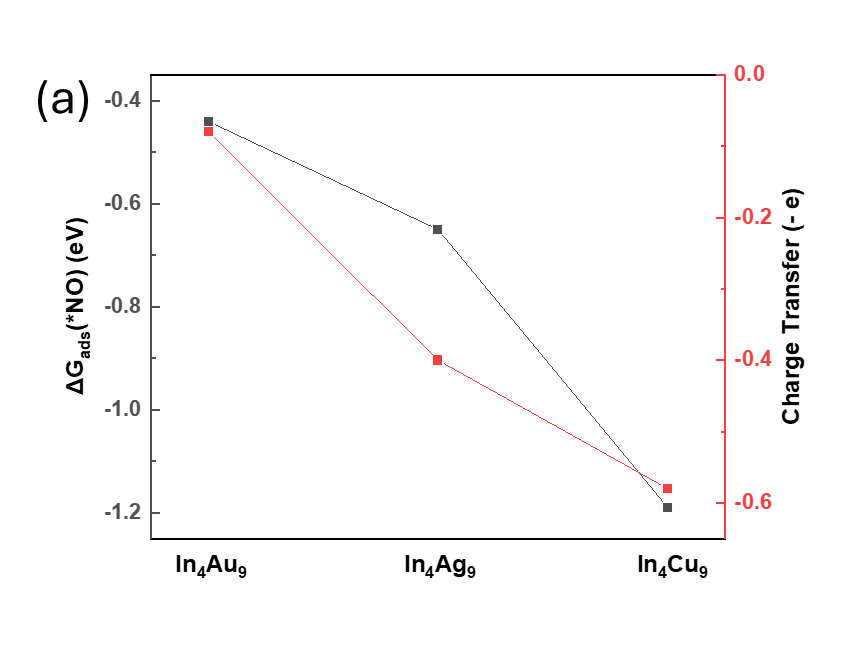


**Figure S5**. The Gibbs free energy changes of NO adsorption vs charge transfer on In_4_Au_9_, In_4_Ag_9_, and In_4_Cu_9_, where the amount of charge transfer is in accordance with NO adsorption free energy.


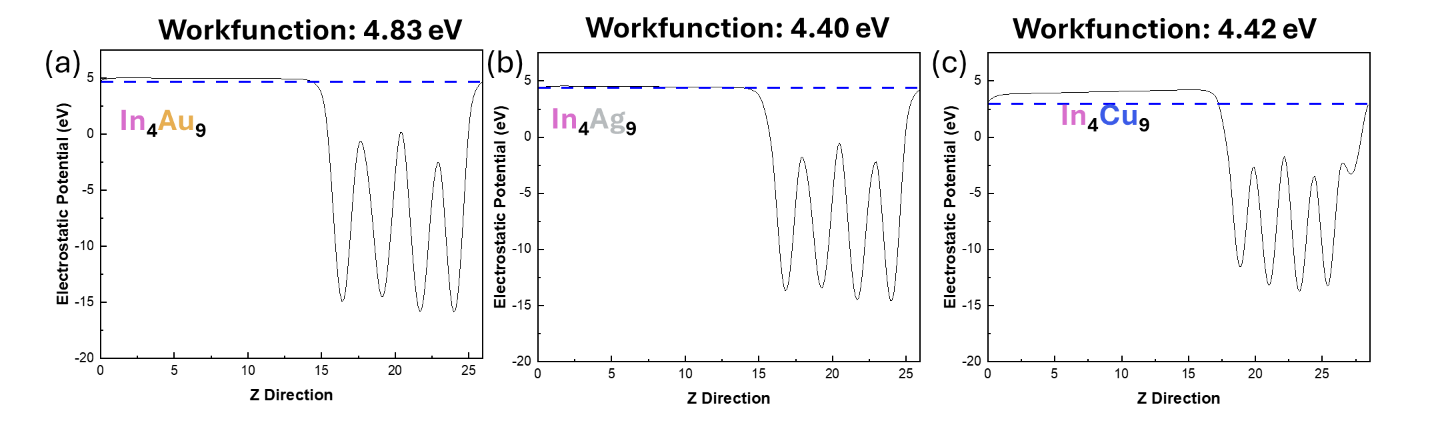


**Figure S6**. The work function of In_4_Au_9_, In_4_Ag_9_, and In_4_Cu_9_.


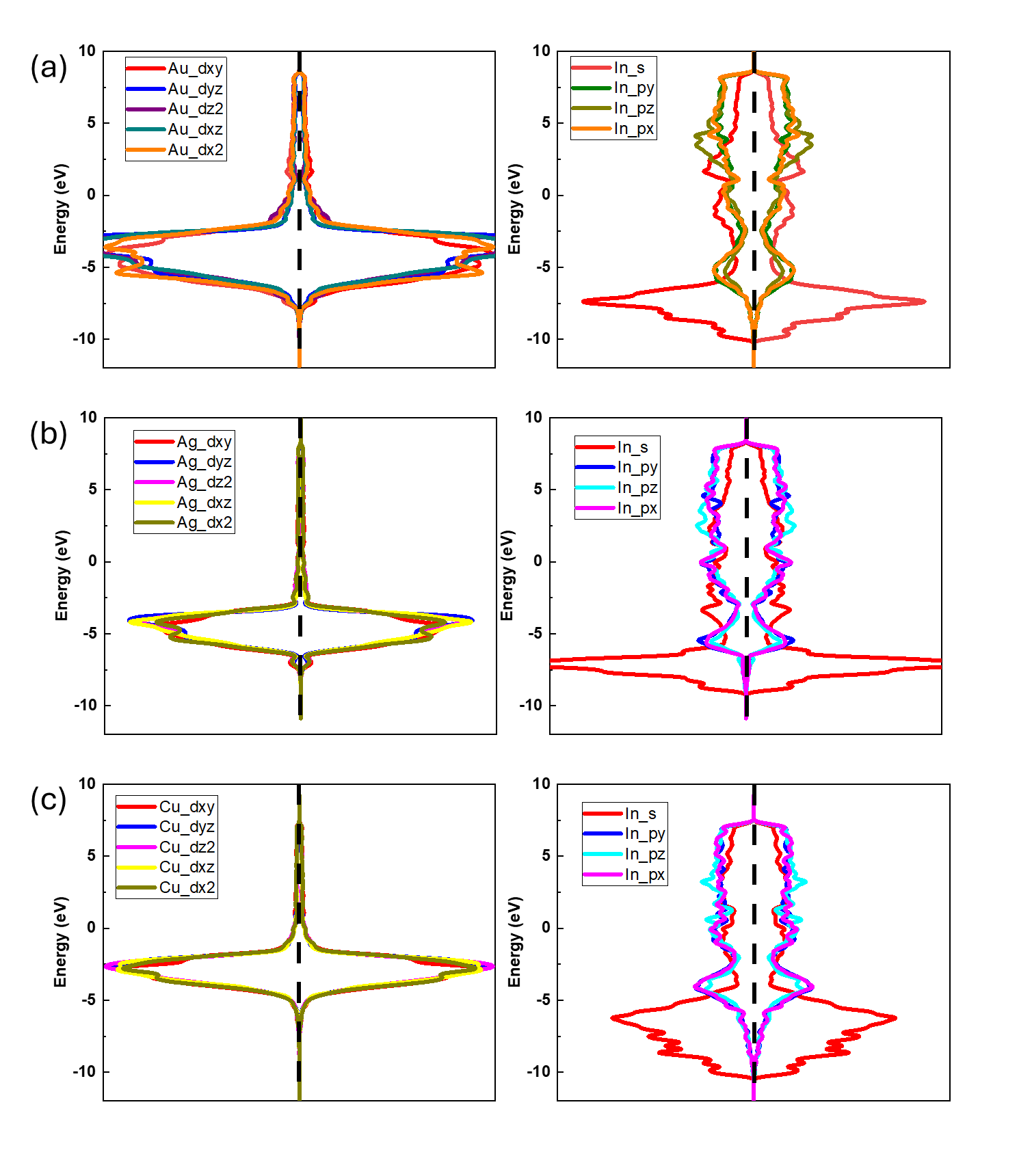


**Figure S7**. (a-c) The PDOS of In_4_Au_9_, In_4_Ag_9_, and In_4_Cu_9_.

**
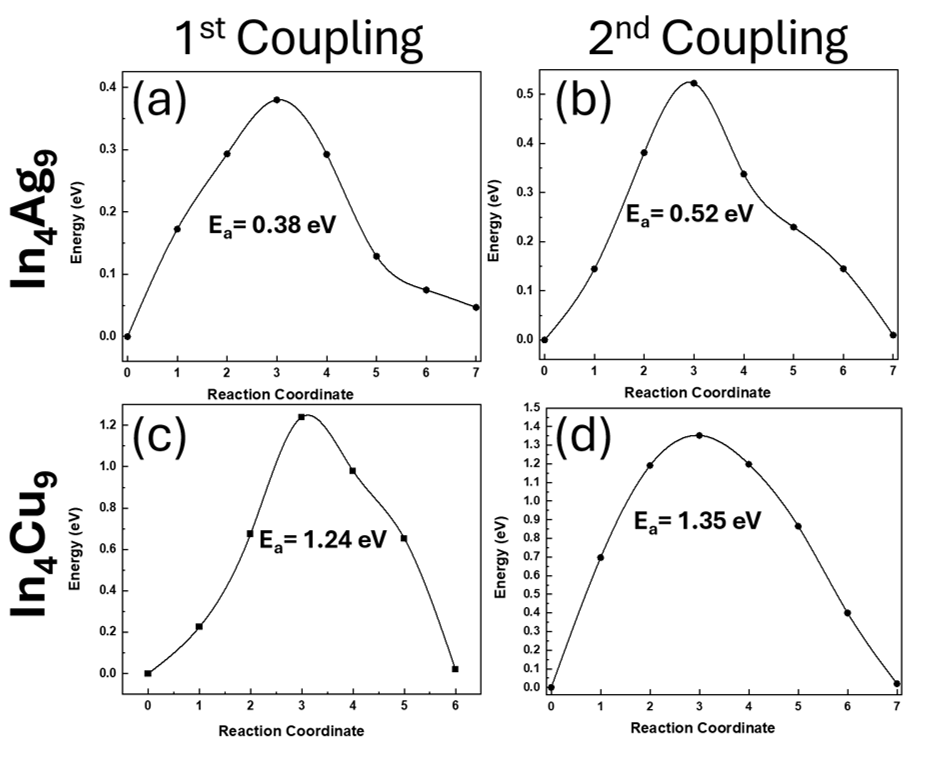
**

**Figure S8**. The energy barrier changes of C-N coupling for In_4_Ag_9_ and In_4_Cu_9_ in the process of urea production.

**
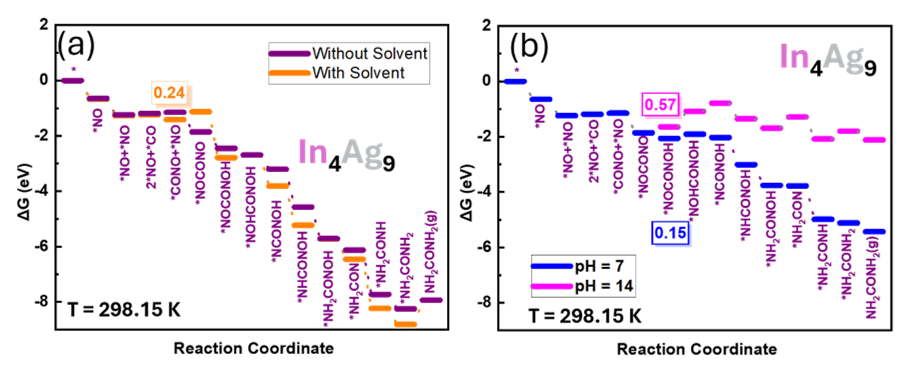
**

**Figure S9**. The Gibbs free energy evolution of In4Ag9 for urea production under the influence of solvent and pH.


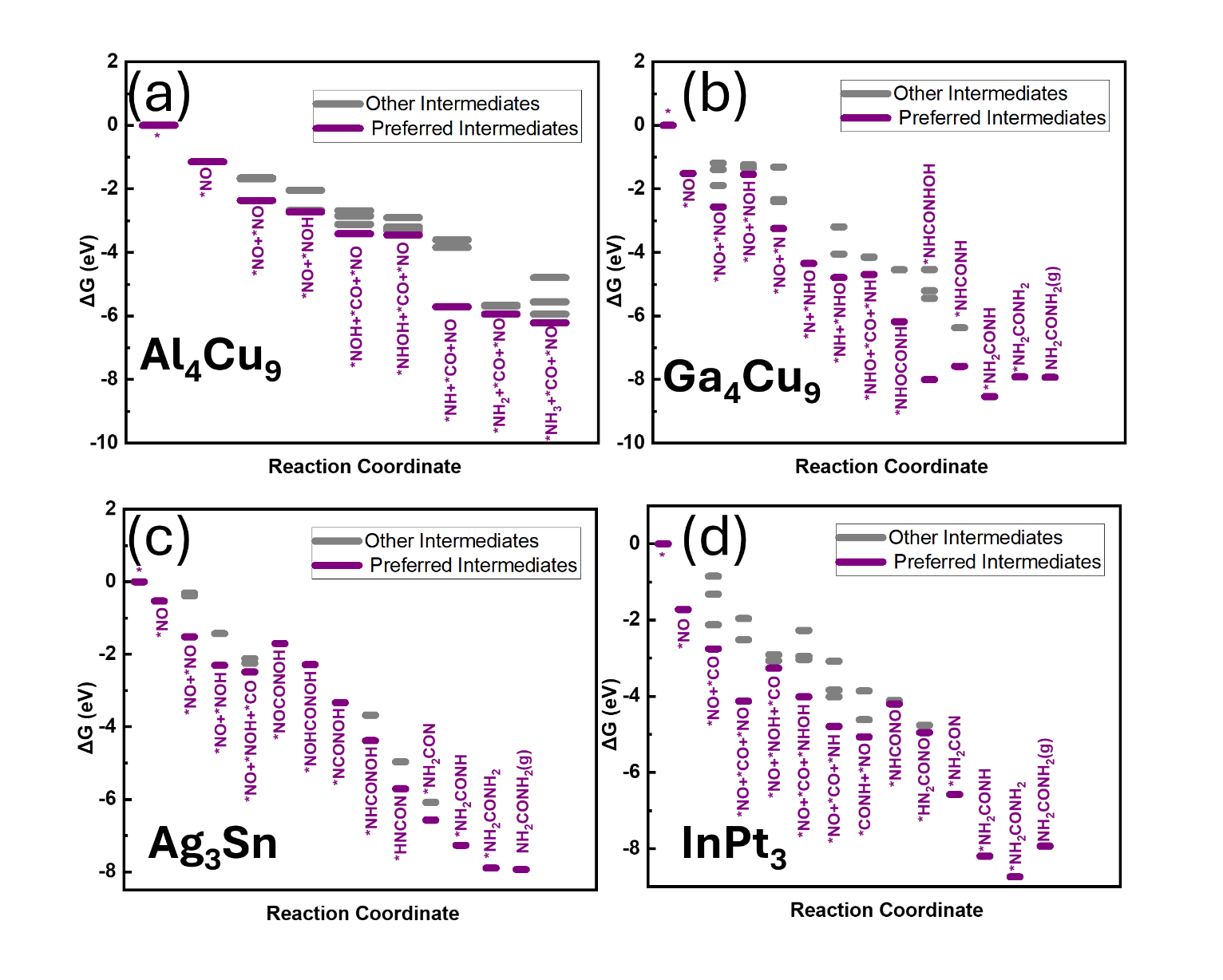


**Figure S10**. (a-d) The Gibbs free energy evolution for Al_4_Cu_9_, Ga_4_Cu_9_, Ag_3_Sn and InPt_3_ for urea production. The thermodynamically unfavourable intermediates are shown in grey in comparison.


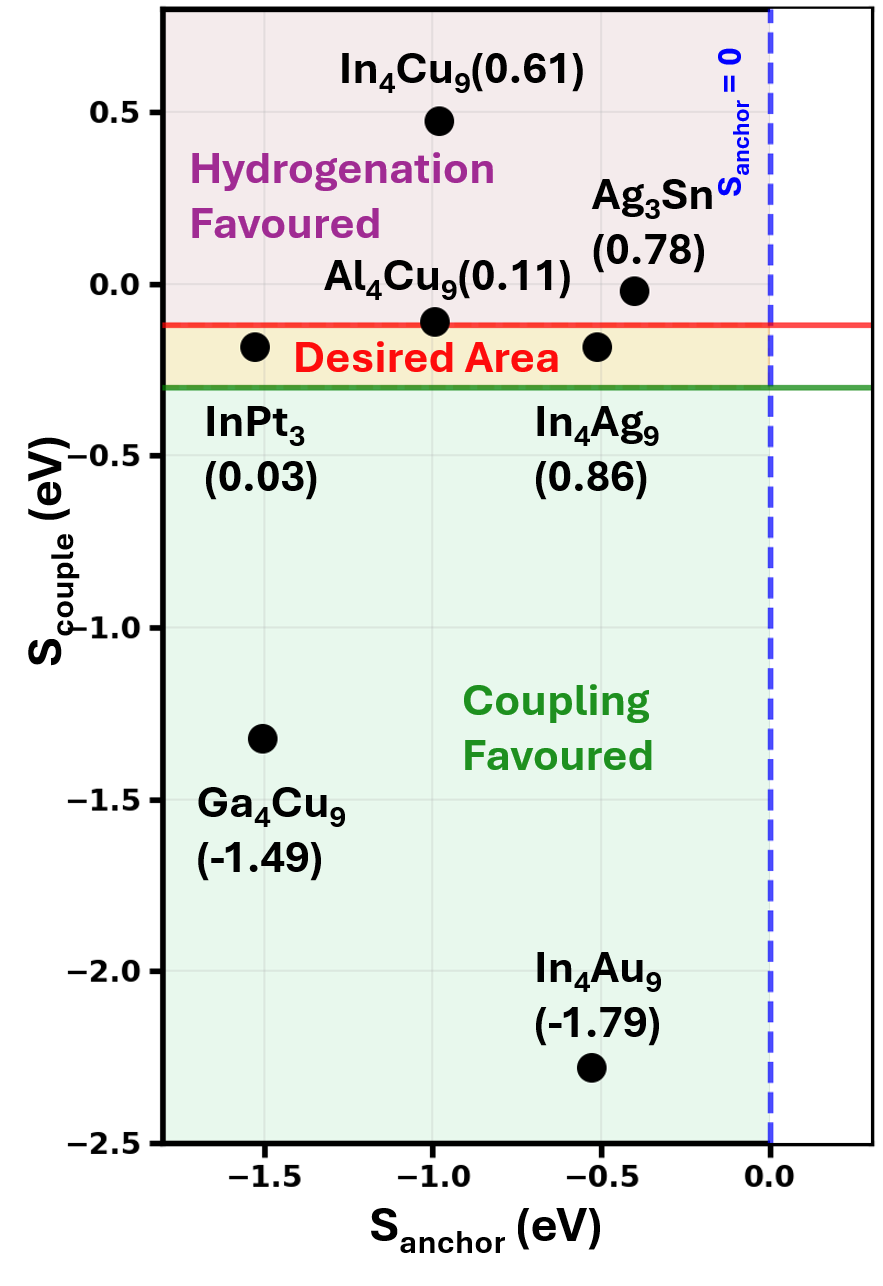


**Figure S11**. The comparison of highest free energy demand for C-N coupling from the upper zone (Hydrogenation Favourable) to the lower zone (Coupling Favourable), where C-N coupling becomes easier from the light pink to the light green zone.


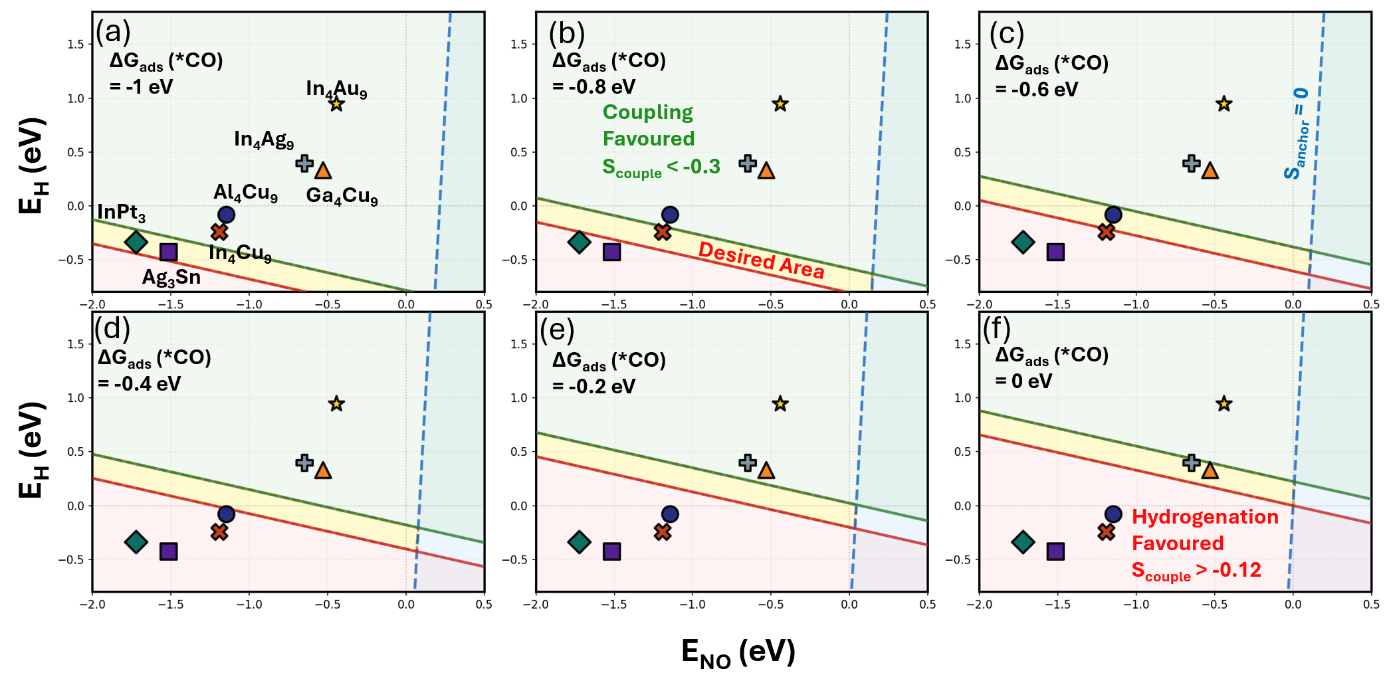


**Figure S12**. (a-f) The interplay of CO and NO adsorption energy with the energy increase of proton from -1eV to 0 eV, where the light yellow indicates the desired area.


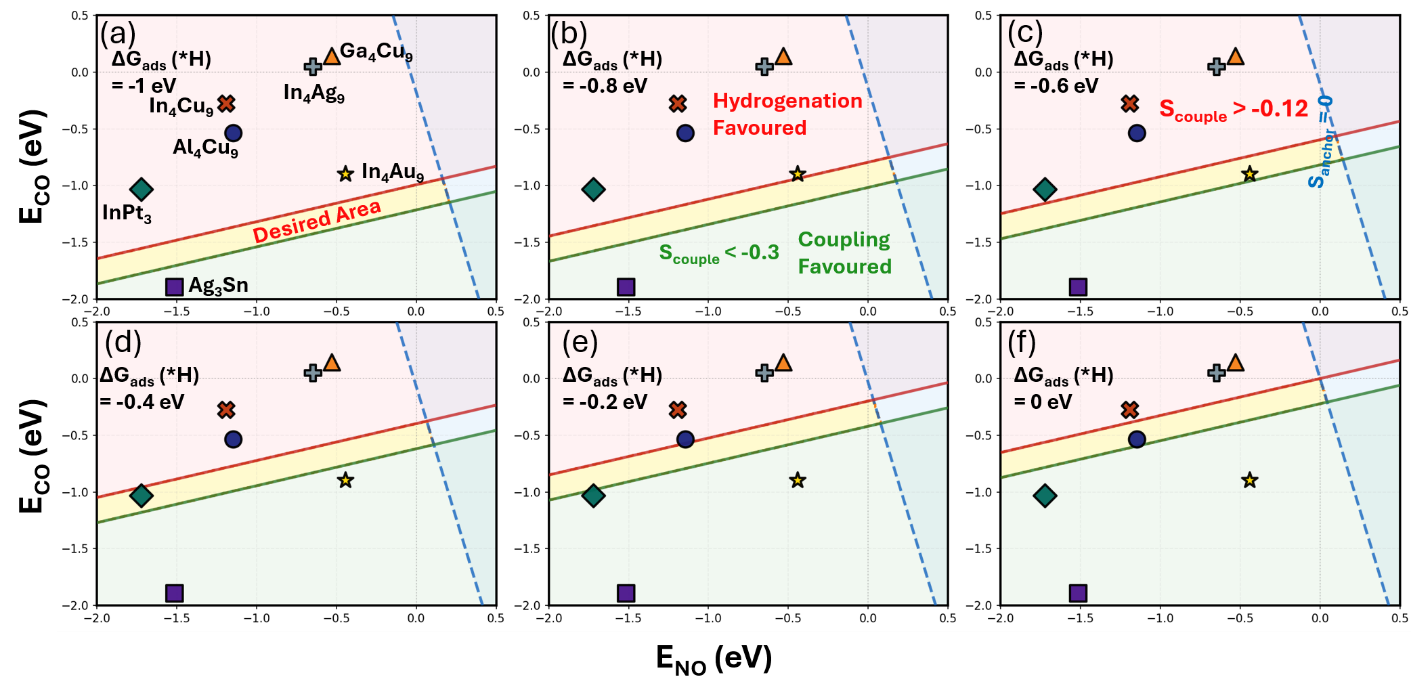


**Figure S13**. (a-f) The interplay of CO and NO adsorption energy with the energy increase of proton from -1eV to 0 eV, where the light yellow indicates the desired area.

**Table S1**. The choice of In_4_M_9_ with crystal information and stable facet based on databases of Materials Project (MP).

| **MP ID** | **Name** | **Hermann Mauguin** | **Crystal System** | **Stable Facet** |
| --- | --- | --- | --- | --- |
| mp-680545 | In_4_Au_9_ | P4̅3m | Cubic | 110 |
| mp-21975 | In_4_Ag_9_ | P4̅3m | Cubic | 110 |
| mp-683917 | In_4_Cu_9_ | P4̅3m | Cubic | 110 |

**Table S2**. The comparison of pairwise competitiveness of In_4_Au_9_ with intermediate, reaction pathways and energy gaps.

| In_4_Au_9_ | Key intermediates | Reaction pathways | Energy gaps (eV) |
| --- | --- | --- | --- |
| Adsorption vs Hydrogenation | *NO | *NO +CO 🡪 *NO+*CO | -0.17 |
|  |  | *NO +NO 🡪 *NO+*NO | -0.89 |
|  |  | *NO + H 🡪 *NOH | 0.32 |
|  |  | *NO + H 🡪 *NHO | -0.01 |
| 1^st^ Coupling vs Hydrogenation | *NO+*NO | *NO+*NO+*CO 🡪 *NO-CO-NO | -0.08 |
|  |  | *NO+*NO +H 🡪 *NO+*NOH | -0.41 |
|  |  | *NO+*NO +H 🡪 *NO+*NHO | 0.39 |
| 2^nd^ Coupling vs Hydrogenation | *NO+*CO-NO | *NO+*NO 🡪 *NO -NO | -2.18 |
|  |  | *NO+*NO + H🡪 *NOH+* NO | -0.48 |
|  |  | *NO+*NO + H🡪 *NHO+*NO | -0.48 |

**Table S3.** The comparison of pairwise competitiveness of In_4_Ag_9_ with intermediate, reaction pathways and energy gaps.

| In_4_Ag_9_ | Key intermediates | Reaction pathways | Energy gaps (eV) |
| --- | --- | --- | --- |
| Adsorption vs Hydrogenation | *NO | *NO +CO 🡪 *NO+*CO | 0.027 |
|  |  | *NO +NO 🡪 *NO+*NO | -0.59 |
|  |  | *NO + H 🡪 *NOH | 0.49 |
|  |  | *NO + H 🡪 *NHO | 0.06 |
|  | *NO+*NO | *NO+*NO+CO 🡪 *NO+*NO + *CO | 0.048 |
|  |  | *NO+*NO + H 🡪 *NOH+*NO | 0.70 |
|  |  | *NO+*NO + H 🡪 *NHO+*NO | 0.06 |
| 1^st^ Coupling vs Hydrogenation | *NO+*NO+*CO | *NO+*NO+*CO 🡪 *NO+*CO-NO | 0.039 |
|  |  | *NO+*NO+*CO +H 🡪 *NO+*NOH+*CO | 0.55 |
|  |  | *NO+*NO+*CO +H 🡪 *NO+*NHO+*CO | 0.12 |
| 2^nd^ Coupling vs Hydrogenation | *NO+*CO-NO | *NO+*CO-NO 🡪 *NO-CO-NO | -0.71 |
|  |  | *NO+*CO-NO + H🡪 *NOH+*CO-NO | 0.10 |
|  |  | *NO+*CO-NO + H🡪 *NHO+*CO-NO | -0.41 |

**Table S4.** The comparison of pairwise competitiveness of In_4_Cu_9_ with intermediate, reaction pathways and energy gaps.

| In_4_Cu_9_ | Key intermediates | Reaction pathways | Energy gaps (eV) |
| --- | --- | --- | --- |
| Adsorption vs Hydrogenation | *NO | *NO +CO 🡪 *NO+*CO | -0.56 |
|  |  | *NO +NO 🡪 *NO+*NO | -1.44 |
|  |  | *NO + H 🡪 *NOH | 0.05 |
|  |  | *NO + H 🡪 *NHO | 0.05 |
|  | *NO+*NO | *NO+*NO+CO 🡪 *NO+*NO + *CO | -0.27 |
|  |  | *NO+*NO + H 🡪 *NOH+*NO | -0.08 |
|  |  | *NO+*NO + H 🡪 *NHO+*NO | 0.38 |
| 1^st^ Coupling vs Hydrogenation | *NH_2_+*N+*CO | *NH_2_+*N+*CO 🡪 *NH_2_-CO+*N | 0.57 |
|  |  | *NH_2_+*N+*CO 🡪 *NH_2_+*CON | -1.74 |
|  |  | *NH_2_+*N+*CO + H🡪 *NH_2_+*CO+*NH | -1.33 |
| 2^nd^ Coupling vs Hydrogenation | *NH_2_+*CO-N | *NH_2_+*CO-N 🡪 *NH_2_-CO-N | 0.61 |
|  |  | *NH_2_+*CO-N+H 🡪 *NH_2_+CO-NH | 0.67 |

**References:**

[1] J. K. Nørskov, J. Rossmeisl, A. Logadottir, L. Lindqvist, J. R. Kitchin, T. Bligaard, H. Jónsson, “Origin of the Overpotential for Oxygen Reduction at a Fuel-Cell Cathode”, *The Journal of Physical Chemistry B*, 108 (2004), 17886-17892, 10.1021/jp047349j.

[2] V. Wang, N. Xu, J.-C. Liu, G. Tang, W.-T. Geng, “VASPKIT: A user-friendly interface facilitating high-throughput computing and analysis using VASP code”, *Computer Physics Communications*, 267 (2021), 108033, <https://doi.org/10.1016/j.cpc.2021.108033>.
